# Severe Congenital Candidiasis due to *Candida dubliniensis* Treated With Fosfluconazole in a Preterm Infant

**DOI:** 10.1155/crpe/7680042

**Published:** 2026-04-27

**Authors:** Hiroyuki Higashiyama, Takeshi Futatani

**Affiliations:** ^1^ Department of Neonatology, Toyama Prefectural Central Hospital, Toyama, Japan, tch.pref.toyama.jp

## Abstract

Congenital candidiasis is a rare but potentially lethal disease in preterm infants. Most cases are caused by *Candida albicans*; to date, there are no reports of *Candida dubliniensis* as the causative organism. Herein, we report a case of severe congenital candidiasis caused by *C*. *dubliniensis* in a very low‐birth‐weight infant. The patient was treated with fosfluconazole intravenously. However, candidiasis infection relapsed on Day 8 of life. Therefore, fosfluconazole administration was switched to 10 mg/kg/day of micafungin for the next 14 days as the inhibitory concentration of micafungin was lower than that of fluconazole. The candidiasis did not relapse again after discontinuation of micafungin. The patient was discharged on Day 59 without apparent sequelae. Some experiments have induced stable fluconazole resistance in vitro in fluconazole‐sensitive *C*. *dubliniensis* strains after exposure to low concentrations of fluconazole. It may be advisable to consider an alternative antifungal drug when *C*. *dubliniensis* is a causative organism.

## 1. Introduction

Congenital candidiasis is a rare disease, with a diffuse transient skin eruption being the only symptom in most term infants. Despite its rarity, this disease is potentially lethal in preterm infants [1]. Most cases of congenital systemic candidiasis and late‐onset invasive candidiasis (IC) in neonatal intensive care units are caused by *Candida albicans*. Only 19 cases of non‐*albicans Candida*–associated congenital systemic candidiasis have been reported to date [2, 3]. *Candida dubliniensis* was first described in 1995 as atypical oral isolates recovered from individuals infected with human immunodeficiency virus. Its phenotypic characteristics are notably similar to that of *C*. *albicans*, making it difficult to distinguish between the two organisms by routine laboratory methods such as optical microscopy and cultures [4]. Reports of late‐onset IC caused by *C*. *dubliniensis* in neonates are increasing in number; however, to the best of our knowledge, there is no report of confirmed congenital cases [5, 6].

Fluconazole is the most commonly used antifungal agent in low‐birth‐weight infants because it has favourable characteristics in terms of activity, safety and compatibility with other commonly used neonatal medications [7]. Additionally, this antifungal is reported to be active against most isolates of *C*. *dubliniensis* in various clinical situations [8–10]. Moran et al. showed that stable fluconazole resistance was induced in vitro in fluconazole‐sensitive *C*. *dubliniensis* strains after exposure to low concentrations of fluconazole [11]. Fosfluconazole, a prodrug of fluconazole converted rapidly by alkaline phosphatase in the body, requires less solution volume compared to fluconazole because of its high solubility. Considering that premature infants have little capacity for fluid administration, it frequently replaces fluconazole in Japanese neonatal intensive care units [12]. Herein, we report a case of congenital candidiasis caused by *C*. *dubliniensis* and treated with fosfluconazole in a preterm infant.

## 2. Case Presentation

**FIGURE 1:**
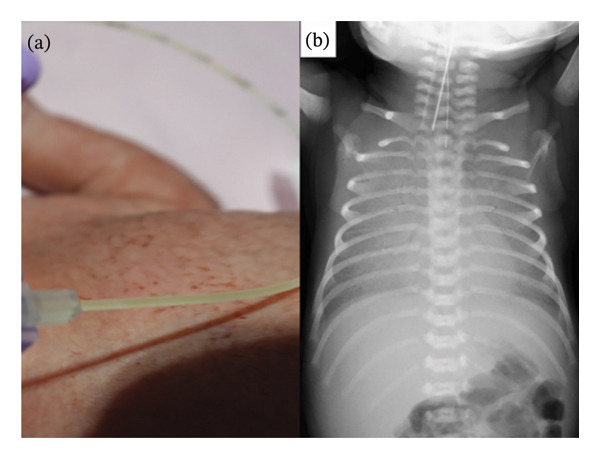
(a) Small, well‐circumscribed red spots on the patient’s trunk. (b) Chest radiograph of the patient on admission to the neonatal intensive care unit. Widespread opacification throughout both lung fields is seen even after lung surfactant had been administrated.

**FIGURE 2:**
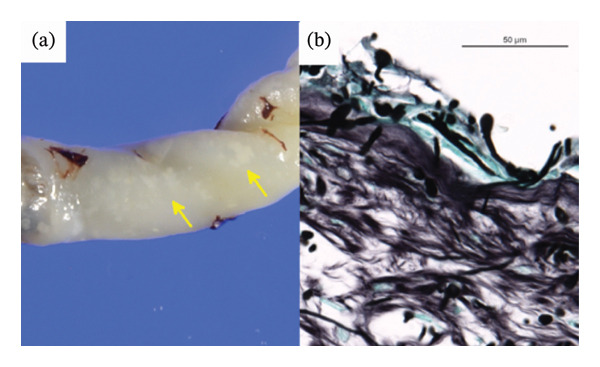
(a) Small, circumscribed yellow‐white nodules on the surface of the umbilical cord (yellow arrows). (b) Microscopic view of a nodule with the Grocott stain. Some filamentous fungi and yeasts are invading beneath the umbilical surface.

The patient was born at a gestational age of 30 weeks due to premature labour. The pregnancy course had been unremarkable until 29 weeks of gestation. The patient’s mother had been initially admitted to another hospital for threatened preterm labour. She had an elevated C‐reactive protein (CRP) level (54 mg/L). *C*. *albicans* was identified in the vaginal discharge of the mother, and the screening test was positive for group B streptococcus. Following these results, the mother was transferred to our hospital. The membranes ruptured prematurely a day before delivery. The female patient weighed 1349 g and presented with respiratory distress. The 1‐min and 5‐min Apgar scores were 7 and 9, respectively. A tracheal tube was placed, and lung surfactants were administered immediately after birth. The patient was then transferred to the neonatal intensive care unit and placed on a ventilator. The patient was afebrile (36.7°C) with a blood pressure of 49/27 mmHg, heart rate of 157 beats/min, respiratory rate of 80 breaths/min and oxygen saturation of 93%. On examination, the patient showed mild subcostal retractions and shallow respirations. Breath sounds were slightly diminished in intensity. An eruption of small, well‐circumscribed, erythematous macules was noted on the patient’s trunk (Figure 1(a)). The remainder of the examination findings were normal for the gestational age. Chest radiography revealed widespread opacification throughout both lung fields even after lung surfactant administration (Figure 1(b)). Laboratory tests showed a white blood cell (WBC) count of 44.1 × 10^9^/L, a platelet (PLT) count of 247 × 10^9^/L, aspartate aminotransferase (AST) of 44 U/L, alanine aminotransferase (ALT) of 6 U/L, blood urea nitrogen (BUN) of 90 mg/L, creatinine (Cr) of 4.5 mg/L, CRP of 3.7 mg/L and glucose of 920 mg/L. Gram‐positive yeasts were identified microscopically in the sample from gastric aspiration. Matrix‐assisted desorption/ionization‐time of flight mass spectrometry (MALDI‐TOF MS) identified *C. dubliniensis* in the gastric aspirates, skin, bronchial secretions and vernix of the patient. Antifungal susceptibility testing was performed using the colourimetric broth microdilution method. The results were interpreted in accordance with the Clinical and Laboratory Standards Institute (CLSI) guidelines. The minimum inhibitory concentrations (MICs) of amphotericin B, fluconazole and micafungin were 0.5, 0.25 and < 0.03 mg/L, respectively. No other organism, including group B streptococcus, was detected from the patient. The umbilical cord showed small circumscribed yellow‐white nodules on its surface (Figure 2(a)). A microscopic view of those areas with the Grocott stain showed that some filamentous fungi and yeasts were infiltrating beneath the umbilical surface (Figure 2(b)). The blood culture taken on Day 10 was negative. The fundus examination on Day 11 was normal.

**FIGURE 3:**
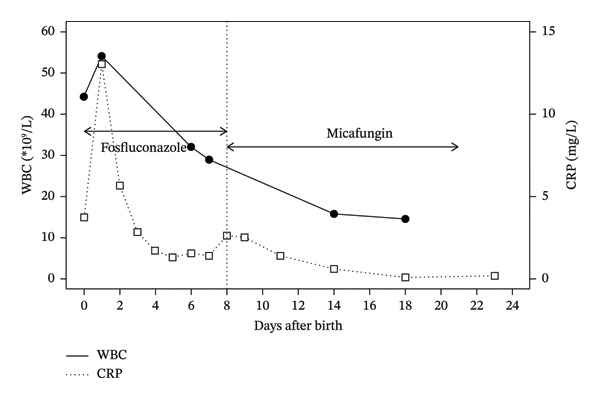
Trend in C‐reactive protein values after birth.

An intrauterine infection, particularly group B streptococcal or *Listeria monocytogenes* infection, was considered as a differential diagnosis based on the mother’s positive screening test result for group B streptococcus and observed skin rash. Infections by other microorganisms, such as herpes simplex and varicella‐zoster virus, may also present with congenital skin rash; however, umbilical cord lesions are seen at the same time in only *Listeria monocytogenes* infections other than congenital candidiasis [1]. As these microorganisms were not detected in microscopic or culture studies, we diagnosed the patient with congenital candidiasis due to *C*. *dubliniensis*. An antimicrobial regimen involving 100 mg/kg/day of ampicillin sodium, 15 mg/kg/day of amikacin sulphate and 7.6 mg/kg/day of fosfluconazole (equivalent to 6 mg/kg of fluconazole) was initiated. The patient was extubated on Day 1 of life. Skin eruption had disappeared by Day 3. Ampicillin sodium and amikacin sulphate were discontinued on Days 3 and 2, respectively, due to confirmation of candidiasis. CRP levels steadily declined from Day 1 to Day 5 (from 13 mg/L on Day 1 to 1.3 mg/L on Day 5), remained stable on Day 6 (1.6 mg/L) and increased on Day 8 (2.6 mg/L) (Figure 3). Laboratory tests on Day 7 revealed a WBC count of 32.1 × 10^9^/L, a PLT count of 562 × 10^9^/L, AST of 35 U/L, ALT of 10 U/L, BUN of 230 mg/L, Cr of 9.8 mg/L, CRP of 1.4 mg/L and glucose of 930 mg/L. Although she was in a stable condition, fosfluconazole was switched to micafungin (10 mg/kg/day) on Day 8 for the next 14 days after obtaining cultures from the central catheter tip and catheterised urine. No organisms were recovered, except for a small amount of *Staphylococcus epidermidis* in the urine. No antibiotics were added. Micafungin had the least value of MIC compared with fluconazole and amphotericin B. The patient achieved a full recovery, and the CRP value did not elevate again (0.2 mg/L on Day 23). We interpreted this elevation in CRP as a potential sign of relapse because marked leucocytosis had persisted until Day 8, when micafungin was initiated; no significant bacterial growth was observed, and both the WBC count and CRP level normalised without the administration of antibiotics. The patient was discharged from the hospital on Day 59. She was able to walk and speak several words at the corrected age of 1 year and 4 months, showing no apparent sequelae.

## 3. Discussion

The only known clinical feature of congenital candidiasis is transient skin rash. The similarity in microbiological phenotypes between *C*. *albicans*, the disease’s most common causative pathogen, and *C*. *dubliniensis* creates challenges in identification through conventional methods [4]. In this case report, *C*. *albicans* was identified in the vaginal discharge of the patient’s mother at the previous hospital. However, in the patient, *C*. *dubliniensis* was confirmed as the causative pathogen using MALDI‐TOF MS, which can rapidly and precisely identify microorganisms including yeasts [13]. To the best of our knowledge, this is the first confirmed case report of congenital systemic candidiasis caused by *C. dubliniensis*.

Fluconazole is commonly used for prophylaxis and treatment of *Candida* species infections in premature infants [7]. We administered 7.6 mg/kg of fosfluconazole (equivalent to 6 mg/kg of fluconazole) every 24 h as the first‐line drug. CRP levels decreased over the next 5 days; however, they increased again on Day 8 of life. In Japan, 6 mg/kg fluconazole every 72 h is recommended as the initial dose for neonatal IC. However, Wade et al. reported that higher doses of 12 mg/kg/day are required to achieve a target area under the concentration curve of more than 0.4 g·h/L at 24 h [14]. The clinical guideline by the Infectious Diseases Society of America in 2016 recommends 12 mg/kg/day of fluconazole as an alternative therapy for IC in neonates [15]. Moran et al. showed that stable fluconazole resistance was induced in vitro in fluconazole‐sensitive *C*. *dubliniensis* strains after exposure to low concentrations of fluconazole [11]. The main mechanism for fluconazole resistance in *C*. *dubliniensis* is similar to that of *C*. *albicans*: an overexpression of major facilitator efflux pumps MDR1 and CDR1 [16]. Both the area under the curve and the susceptibility to fluconazole at the time of CRP reelevation were not examined in our case; therefore, the reason for the increase remains unclear. Nevertheless, it is recommended to administer a sufficient amount of fluconazole according to pharmacodynamics or to initiate alternative drugs, such as micafungin or amphotericin B, when *C*. *dubliniensis* is confirmed as the causative pathogen.

Micafungin was selected not only because it exhibited the lowest MIC among the isolates compared with amphotericin B and fluconazole but also due to its distinct mechanism of action. It inhibits cell‐wall synthesis rather than targeting the cell membrane, as fluconazole does. Although data remain limited, its efficacy and safety in neonates with IC have been demonstrated in Phase 2 (NCT03421002) and Phase 3 (NCT00815516) clinical trials, showing comparable outcomes to amphotericin B [17, 18]. Furthermore, Ibrahim et al. reported that micafungin treatment in neonates achieved a high success rate with fewer complications compared with amphotericin B [19].

In conclusion, we report a case of congenital systemic candidiasis caused by *C. dubliniensis* in a preterm infant. *C. dubliniensis* has a higher propensity to develop stable fluconazole resistance through the rapid upregulation of efflux pumps when exposed to subtherapeutic concentrations. Therefore, clinicians should ensure adequate fluconazole dosing or consider micafungin as a preferred treatment option when *C. dubliniensis* is identified, particularly in cases where clinical improvement is suboptimal.

## Author Contributions

Hiroyuki Higashiyama drafted the initial manuscript and reviewed and revised the manuscript. Takeshi Futatani critically reviewed the manuscript for important intellectual content.

## Funding

This work did not receive any specific grant from funding agencies in the public, commercial or not‐for‐profit sectors.

## Disclosure

All authors approved the final manuscript as submitted and agree to be accountable for all aspects of the work.

## Ethics Statement

This study was conducted in accordance with the Declaration of Helsinki (1964). Written informed consent was obtained from the patient’s parent for the patient’s treatment and the publication of this case report.

## Conflicts of Interest

The authors declare no conflicts of interest.

## Data Availability

The data that support the findings of this study are available on request from the corresponding author. The data are not publicly available due to privacy or ethical restrictions.
